# Review of the Clinical Experiences of Feeding Infants Formula Containing the Human Milk Oligosaccharide 2′-Fucosyllactose

**DOI:** 10.3390/nu10101346

**Published:** 2018-09-21

**Authors:** Elizabeth J. Reverri, Amy A. Devitt, Janice A. Kajzer, Geraldine E. Baggs, Marlene W. Borschel

**Affiliations:** Abbott Nutrition, Abbott Laboratories, Columbus, OH 43219, USA; elizabeth.reverri@abbott.com (E.J.R); amy.devitt-maicher@abbott.com (A.A.D.); janice.kajzer@abbott.com (J.A.K.); geraldine.baggs@abbott.com (G.E.B.)

**Keywords:** 2′-fucosyllactose, 2′-FL, human milk oligosaccharide, HMO, infant formula, infant growth, infant, gastrointestinal tolerance, respiratory infection, partially hydrolyzed formula

## Abstract

Human milk oligosaccharides (HMOs) are the third most abundant solid component in human milk after lactose and lipids. Preclinical research has demonstrated that HMOs and specifically 2′-fucosyllactose (2′-FL) are more than a prebiotic and have multiple functions, including immune, gut, and cognition benefits. Previously, human milk has been the only source for significant levels of HMOs. The most abundant HMO in most mothers’ breast milk is 2′-FL. Recently, 2′-FL has been synthesized and shown to be structurally identical to the 2′-FL found in human milk. 2′-FL HMO is now available in some commercial infant formulas. The purpose of this narrative review was to summarize the clinical experiences of feeding infant formula supplemented with the HMO, 2′-FL. Most of these studies investigated standard intact milk protein-based infant formulas containing 2′-FL, and one evaluated a partially hydrolyzed whey-based formula. Collectively, these clinical experiences demonstrated that 2′-FL being added to infant formula was safe, well-tolerated, and absorbed and excreted with similar efficiency to 2′-FL in human milk. Further, infants that were fed formula with 2′-FL had immune benefits, fewer parent-reported respiratory infections, and improved symptoms of formula intolerance. Ultimately, infant formula with 2′-FL supports immune and gut health and is closer compositionally and functionally to human milk.

## 1. Introduction

Human milk oligosaccharides (HMOs) are unique, bioactive carbohydrates [1]. HMOs are the third most abundant solid component in human milk after lactose and lipids [2]. Of the more than 100 different HMOs that have been identified in human milk [1], less than 50 are present in significant amounts [3]. The most abundant HMO in the majority of mothers’ breast milk is 2′-fucosyllactose (2′-FL) [4,5], a trisaccharide consisting of glucose, galactose, and fucose [4]. To date, there have been two large, international analyses of HMOs in human milk [4,5]. The first comprehensive analysis of HMOs from human milk in approximately 400 lactating women from 10 countries found that 85% of human milk samples had detectable 2′-FL at concentrations of 0.06–4.65 g 2′-FL/L [4]. The second study found similar results from 410 lactating women from 11 international cohorts with 65–98% of human milk samples having 2′-FL with mean concentrations ranging from 0.702–3.440 g 2′-FL/L [5].

HMOs resist digestion in the upper gastrointestinal (GI) tract. Evidence suggests that the majority of HMOs reach the large intestine intact [6,7]. A small portion of ingested HMOs are absorbed intact into the circulation [8] and are excreted in urine [9], which may explain the systemic benefits of HMOs [8,10]. For example, a key window of immune development occurs during the first few weeks of life when innate immune cells peak in the circulation [11,12,13,14]. Furthermore, because ~70% of immune cells reside in the digestive tract [15], they may interact directly with HMOs that are consumed by infants [2]. HMOs have been studied observationally from human milk for their immune benefits. In a study of 93 mother-infant pairs, the mother’s breast milk was analyzed for HMOs between one and five weeks postpartum and infant feeding and diarrhea data were collected for two years. Infants whose mother’s breast milk had low concentrations of 2′-FL and another 2-linked fucosyloligosaccharide HMO, lacto-*N*-difucohexaose, had significantly higher rates of *Campylobacter* diarrhea and calicivirus diarrhea, respectively, than infants who were fed with their mother’s breast milk with higher levels of these HMOs [16]. Further, a pilot study evaluated the relationship between the HMO, lacto-*N*-fucopentaose II, which was used as a surrogate measure for the levels of HMOs in human milk, and subsequent disease in 49 mother-infant pairs. Infants who experienced respiratory problems or GI problems by six and 12 weeks of age had mother’s breast milk containing significantly lower levels of the HMO in milk samples that were collected at two weeks of age, compared to infants who had no respiratory or GI problems [17].

Because 2′-FL is the most abundant HMO that is present in the milk of most lactating women, it has been the most studied HMO in regard to its potential systemic effects. Preclinical research shows that 2′-FL has multiple functions including: acting as a prebiotic [18,19,20,21,22,23,24], protecting against infections and inflammation [25,26], modulating the immune system [27,28,29,30,31,32,33,34], supporting brain development [35,36,37,38,39], and reducing the risk of necrotizing enterocolitis [40,41]. Synthesized 2′-FL is structurally identical to the 2′-FL in human milk. The recent availability of synthesized 2′-FL is important because HMOs were previously only found at significant levels in human milk, however they are now available in some commercial infant formulas. Further, several 2′-FL ingredients have been the subject of Generally Recognized As Safe (GRAS) notifications and the US Food and Drug Administration has no questions on the proposed addition to infant formula [42]. Internationally, the European Union has approved the use of 2′-FL in infant formula [43]. Likewise, countries that refer to the European authorization will also adopt the safe addition of 2′-FL to infant formulas.

Thus, the purpose of this review was to summarize the clinical experiences of feeding formulas containing the HMO, 2′-FL, in healthy infants.

## 2. Methods

The purpose of this narrative review was to summarize the clinical experiences of feeding formulas containing only the HMO, 2′-FL, in healthy infants. Our criterion was to search for studies in which infant formula containing 2′-FL HMO was fed to infants. The databases that were searched were Allied & Complementary Medicine™, Analytical Abstracts, BIOSIS Previews^®^, Embase^®^, EMCare^®^, FSTA^®^, MEDLINE^®^, and ToxFile^®^. The search terms included 2′-FL HMO and 2′FL and human milk oligosaccharide or HMO and infant formula or formula.

## 3. Results

In this narrative review, publications were included that only studied the HMO, 2′-FL [10,44], along with two clinical experiences that were previously presented as abstracts [45] or unpublished data that were conducted by Abbott Nutrition (Table 1). A systematic review was not conducted due to the limited number of studies that had heterogenous outcomes.

### 3.1. Growth and Tolerance of Infants Fed Milk-Based Formula with 2′-FL

The first clinical study [44] to investigate 2′-FL in infant formula was a prospective, randomized, controlled, growth, and tolerance study that was conducted in healthy term infants. Infants were enrolled by 5 days of life (*n* = 420). There were four groups, including three randomized formula groups and a breastfed (BF) reference group. The three study formulas included a control formula (CF) without added HMO and two study formulas differing in the amount of 2′-FL: 0.2 g 2′-FL/L versus 1.0 g 2′-FL/L (Abbott Nutrition, Columbus, OH, USA), however all three formulas contained the same amount of total oligosaccharides at 2.4 g/L. The amount of 2′-FL was chosen to be within the range of 2′-FL in human milk. All formula-fed infants were exclusively fed the assigned formula for the duration of the study. The primary outcome was weight gain from 14–119 days and this was not significantly different among the formula groups or the BF reference group. Similarly, tolerance measures (including stool frequency, stool consistency, incidence of spit-up, and vomit associated with feedings) did not differ among the formula groups [44].

As expected, 2′-FL uptake, which was demonstrated by plasma levels, was greatest in the BF group followed by the 1.0 g 2′-FL/L containing formula and then the 0.2 g 2′-FL/L formula group. No 2′-FL was detected in the plasma of the CF fed infants. Despite this difference, relative absorption and excretion (ratio of the amount in circulation or excretion to the concentration that was fed) was comparable and did not significantly differ among the BF and the two 2′-FL containing formula groups. This finding demonstrated that 2′-FL was present systemically in the plasma and urine. Adverse events (AE) were parent reported and physician confirmed. Interestingly, the CF group had significantly higher reports of eczema than the two 2′-FL containing formula groups. Additionally, there were differences noted in the Infections and Infestations System Organ Class (SOC), with both the CF group and the group that was fed formula with 1.0 g 2′-FL/L having more events compared to the group that was fed formula with 0.2 g 2′-FL/L [44]; however, there were no significant differences among the study groups for any specific preferred term (PT). Overall, the 2′-FL containing formulas were effective at maintaining appropriate growth and tolerance and had similar 2′-FL uptake. Also, no safety concerns were observed [45]. Marriage et al. [44] was the first published clinical study of an infant formula with 2′-FL.

### 3.2. Inflammatory Cytokines of Infants Fed Milk-Based Formula with 2′-FL

Goehring et al. [10] utilized blood samples that were obtained from a subset of the infants from the Marriage et al. study [44] described above (*n* = 201). At six weeks of age, blood samples were drawn for markers of immune function. A total of 10 plasma inflammatory cytokines were measured and five were significantly higher in the CF group than both the BF and the 2′-FL containing formula groups: interleukin (IL)-1ra, IL-1α, IL-1β, IL-6, and tumor necrosis factor (TNF)-α. There were no differences in the plasma concentrations of any of the 10 plasma inflammatory cytokines between the BF infants and the infants that were fed formulas with 2′-FL, demonstrating that the addition of 2′-FL resulted in lower levels of multiple cytokines, similar to the levels in the BF infants [10].

Additionally, peripheral blood mononuclear cells (PBMCs) were isolated from the infant’s blood for cellular phenotyping. The PBMCs were stimulated ex vivo with respiratory syncytial virus (RSV) and resulted in significantly higher levels of two cytokines (TNF-α and IL-6) by the CF group compared to the 0.2 g 2′-FL/L formula group and similar levels of these two cytokines between the BF and 0.2 g 2′-FL/L formula groups. The formula with 1.0 g 2′-FL/L was not different from the CF, 0.2 g 2′-FL/L, or the BF groups [10]. Six weeks is a meaningful time to study in terms of HMOs and immune health because the first few weeks of life is when innate immune cells peak in the circulation [11,12,13,14]. These two cytokines may be clinically important because their expression has been associated with the severity of RSV infection [46]. Overall, the circulating plasma inflammatory cytokine profiles and RSV-induced cytokine profiles of the infants that were fed either formula with 2′-FL were similar to those of the BF group [10]. For the first time, the effect of 2′-FL on markers of immune function was investigated in formula fed infants [44].

### 3.3. Gastrointestinal Tolerance of Infants Fed Milk-Based Formula with 2′-FL

A prospective, randomized, multi-center, double-blinded, controlled, tolerance trial was conducted in healthy term infants. The results of this study were previously published as an abstract [45], however further information and discussion are included in this narrative review. The study assessed GI tolerance of infants who were fed formula that was supplemented with 0.2 g 2′-FL/L and 2.0 g scFOS/L (Abbott Nutrition, Columbus, OH, USA), compared to a CF without oligosaccharides and a BF reference group. Infants were recruited from nine sites in the US from January to June, 2015. Written informed consent was provided by parents prior to enrollment. The study was conducted in accordance with the protocol and all applicable regulations, including Good Clinical Practices and the ethical principles originating from the Declaration of Helsinki (ClinicalTrials.gov Identifier NCT02322138). Eligible infants were singletons between 0–8 days of age from a full-term birth with a gestational age of 37–42 weeks and a birth weight ≥2490 g. Exclusionary criteria included adverse maternal, fetal, or infant medical history that may have had potential effects on tolerance, growth, and/or development. Physicians/parents agreed to discontinue medications that may have affected GI tolerance prior to enrollment, including over-the-counter medications, herbal preparations, probiotics, or rehydration fluids. During the study, formula fed infants did not receive >8 fl oz of an alternate feeding (breast milk or formula other than their assigned study formula) per week and the BF infants did not receive >8 fl oz of infant formula per week. Both formulas were standard intact milk protein-based infant formulas, had the same nutrient composition except for oligosaccharide content, and were clinically labelled to mask the identity of the study formulas.

The two formula groups were compared with a BF reference group. The infants were exclusively fed their assigned formula or BF *ad libitum* as the sole source of nutrition throughout the duration of the study. Daily stool records were maintained by the parents throughout the study. Daily intake records were maintained by the parents starting with the first feeding after the enrollment through 14 days of age and for three days before the visit at 35 days of age. Mean rank stool consistency (MRSC) was calculated from the stool records (1 = watery, 2 = loose/mushy, 3 = soft, 4 = formed, and 5 = hard) by taking the mean of the daily means per infant over the study period. Study visits were scheduled at enrollment, 14 ± 3 days of age, and 35 ± 3 days of age. The primary variable was average MRSC, a standard procedure to measure tolerance, from enrollment to 35 days of age. Stool number, intake (formula intake volume, number of feedings, and percent of feedings with spit-up/vomit within one hour of feeding), anthropometric measures (including weight and length), and AEs were also assessed. A sample size of 62 (31 infants per formula group) had 80% power to detect a difference of 0.40, with a common standard deviation of 0.55 (effect size of 0.73) in the average MRSC between the two formula groups using a two-sided 0.05 level *t*-test. Assuming a 25% attrition rate, an enrollment of 84 infants for the formula groups was targeted. In addition, 42 infants in a BF reference cohort were enrolled. The Power procedure from SAS^®^ Version 9.2 was used for sample size estimation. Reported results are from the protocol evaluable analysis. The achieved power given 35 evaluable infants fed 2′-FL and 30 on the CF with MRSC by the end of the study was 83%. Continuous variables were analyzed by fitting analysis of variance, analysis of covariance, or repeated measures models. Categorical variables were tested using Cochran-Mantel-Haenszel tests controlling for study center or Fisher’s exact tests. *p* < 0.05 was considered statistically significant.

A total of 131 infants were enrolled into the clinical trial and 119 infants completed the study duration, with 41 in the formula with 2′-FL group, 36 infants in the CF group, and 42 infants in the BF group. There were no statistically significant differences in the demographic characteristics among the three groups for sex, ethnicity, race, gestational age, weight, length, or age at enrollment. There were also no differences in the anthropometric measures among the groups at enrollment or through 35 days of age. In addition, there were no differences in average MRSC among the three feeding groups from enrollment to 35 days of age. There was a significant difference in MRSC between the CF and BF groups (2.41 ± 0.09 and 2.07 ± 0.08, respectively, *p* = 0.0409) from enrollment to 14 days of age, however there were no significant differences between the CF and 2′-FL groups (2.31 ± 0.10) or the 2′-FL and BF groups. From enrollment to 35 days of age, there were no differences in the average number of stools per day between the formula groups, and the average number of stools per day for the BF group (5.5 ± 0.4) was significantly greater than the formula with 2′-FL (1.9 ± 0.2, *p* < 0.0001) and the CF (2.1 ± 0.2, *p* < 0.0001) groups. From enrollment to 35 days of age, there were no differences between the formula groups for average volume of study formula intake, average number of study formula feedings per day, or the percent of feedings with spit-up/vomit associated with feedings. No safety concerns were identified with the study formulas (data not shown).

This 2′-FL clinical study evaluated the GI tolerance of infants that were fed formulas with and without 2′-FL and scFOS, compared to a BF reference group. No clinically significant differences were found among the three groups from enrollment to 35 days of age for stool consistency, formula intake, anthropometric measures, and percent feedings with spit-up/vomit associated with feeding. As expected, BF infants had a greater number of stools per day. Formula with 2′-FL and scFOS was safe and well tolerated in infants, as evidenced by stool consistency, formula intake, percent feedings with spit-up/vomit, and reported AEs like those of the infants who were fed formula without oligosaccharides or those of the BF infants.

### 3.4. Clinical Feeding Experience of Infants Fed a Partially Hydrolyzed Whey-Based Formula with 2′-FL

A clinical feeding experience study was recently conducted to assess the effects of switching to a partially hydrolyzed whey-based formula (PHF) supplemented with 2′-FL on symptoms of formula intolerance. The study design was a prospective, multi-center, single-arm study investigating infants fed a PHF with 0.2 g 2′-FL/L and 1.8 g scFOS/L (Abbott Nutrition, Columbus, OH, USA). The study formula was a low lactose (<2 g/L) formulation and was clinically labelled to mask the identity of the formula. Infants were recruited from 11 sites in the US from May 2016 to May 2017. Informed consent was obtained prior to enrollment into the study. A central institutional review board approved the study and the study was registered at ClinicalTrials.gov Identifier NCT02757924. At enrollment, the parents completed a Baseline Tolerance Assessment questionnaire, similar to that used in a previous study of formula switching [47], that assessed infant symptoms of formula intolerance over the previous three days. Fussiness was defined as general irritability, discontentment, or discomfort that was difficult to soothe, while gassiness was defined as burping, passing gas, bloating, and abdominal pain. Symptoms of formula intolerance for the severity of fussiness and gassiness were rated on a numbered scale ranging from “not at all” to “extremely/excessive”. Infants were eligible for the clinical feeding experience study if they were identified by parents as “very fussy” or “extremely fussy” at enrollment. Inclusion criteria were healthy infants, gestational age between 37–42 weeks, a birth weight of ≥2490 g, singleton birth, 7–42 days of age at entry, and exclusively formula fed. Exclusion criteria were prior consumption of formula with HMO, use of antibiotics in the previous seven days, use of medications that might affect GI tolerance, participation in another study, and mother’s intent to use breast milk.

Infants received the PHF with 2′-FL as a sole source nutrition *ad libitum* throughout the study period. Symptoms of formula intolerance were assessed by parents using a daily tolerance diary for one month that included the severity of fussiness, gassiness severity, number of spit-ups associated with feeding (within one hour), and hours of crying. Study visits occurred at the day of enrollment and on days 7 and 28 of the study. The primary variable was change in fussiness severity from baseline compared to the first full day (day 1) of study formula feeding. Secondary variables were change in gassiness severity, number of spit-ups, and hours of crying; anthropometrics and AEs were also collected. Based on a 30% attrition rate, the target enrollment was 65 infants for 80% power to detect an improvement in fussiness severity in 70% of the infants versus 50% (assumed rate of placebo effect) after one day. The observed attrition for the primary outcome was 20%, decreasing the required enrollment to 57 infants. The achieved power given 47 evaluable infants on day one was 87%. Symptoms of formula intolerance were analyzed as change from baseline using the Wilcoxon signed rank test performed using PROC NPAR1WAY (SAS^®^ Version 9.4, Cary, NC, USA). Statistical significance was considered with a *p* < 0.05.

A total of 59 infants were enrolled in the clinical feeding experience study. Forty-seven were evaluable on day 1 and 32 were evaluable at day 28. The mean age (±SEM) of enrollment was 25.7 ± 1.4 days of age. When switched to the PHF with 2′-FL, 63.8% of infants (95% confidence interval: 50.1% to 77.6%) showed an improvement in fussiness symptoms after one day of feeding. The median reduction in the severity of fussiness was statistically significant after one day (*p* < 0.0001). The severity of fussiness continued to decrease throughout the duration of the study. Statistically significant improvements were also demonstrated with the severity of gassiness, number of spit-ups, and hours of crying after one day, and these improvements were maintained throughout the study period. Of note, infants had statistically significant (median 22%, *p* < 0.0001) less colicky symptoms (the combination of fussiness, gassiness, and crying) in one day, 30% less in two days, 33% less in just one week, and 40% less in 28 days after the initiation of feeding. Growth (weight in kilograms and length in centimeters) was normal, as expected from a previous study with this same PHF without HMO [48]. AE reporting found no safety concerns.

This infant feeding study was the first to investigate a PHF supplemented with 2′-FL for symptoms of formula intolerance. This PHF differed from a standard intact protein formula because it had low lactose content and the protein was partially hydrolyzed. Overall, the formula was safe and was well tolerated by the infants, similar to the results that were reported by Marriage et al. [44] and Kajzer et al. [45]. Additionally, all four symptoms of formula intolerance (fussiness severity, gassiness severity, number of spit-ups, and hours of crying) were reduced after one day and these reductions were sustained throughout the study period. The results from the current study were similar to those of Berseth et al. [47], which investigated a formula switch during a 28-day feeding trial of either a soy-based formula or a cow’s milk protein-based partially hydrolyzed, reduced-lactose formula in infants that were perceived by parents to be very or extremely fussy. Berseth et al. found that switching to either formula from a cow’s milk protein-based formula improved symptoms of formula intolerance [47].

Switching infant formulas is very common in early infancy. Nevo et al. [49] identified factors leading to formula switches using a parent questionnaire in 200 infants. They found that 47% of healthy term infants had a formula switch within the first six months of life and most of these formula switches were to another cow’s milk-based formula. The main reasons for the formula switch were for regurgitation/vomiting and restlessness. Typically, the formula switches were made without consulting a pediatrician. Although these results were from just one study, it provides some scientific insight into formula switching [49]. Parental impressions of infant behavior change from prior to receiving the formula to after switching to the formula suggested that most infants experienced reduced formula intolerance symptoms and symptom severity when receiving the study formula. Overall, these results suggest that a PHF with 2′-FL, for the first time, was safe and well-tolerated by healthy term, fussy infants.

### 3.5. Post-Hoc Analysis of Adverse Events

Published literature has reported immune benefits of HMOs [25,29,30,50] along with the clinical findings by Goehring et al. [10] and Marriage et al. [44]. There were differences noted in the Infections and Infestations SOC, with the CF group having more subjects compared to the group who were fed formula with 0.2 g 2′-FL/L (21% vs. 10%) [44]; however, there were no significant differences among the study groups for any specific PT. This led to the post-hoc analysis of CF and the 0.2 g 2′-FL/L formula (*n* = 205). PTs were grouped together to form a respiratory tract infection cluster that included: bronchitis, bronchiolitis, bronco-pneumonia, pneumonia, croup infection, nasopharyngitis, sinusitis, and upper respiratory tract infection.

The formula with 2′-FL and CF groups were compared using Fisher’s exact test. Consistent with the original AE analysis [44], a *p* ≤ 0.05 was considered statistically significant. Effect sizes that were expressed as risk difference and the corresponding 95% Wilson confidence interval for the risk difference were reported. Significantly fewer infants had parent reported AEs in the Respiratory Tract Infections AE cluster from infants consuming the formula with 0.2 g 2′-FL/L compared to the CF (4% vs. 12%; *p* = 0.0383; effect size = −8.04% [95% CI: −16.76%, −0.20%]). In this post-hoc analysis, the formula with 0.2 g 2′-FL/L was associated with significantly fewer respiratory infections, compared to the CF.

## 4. Conclusions

In conclusion, this narrative review found that there are several clinical studies reporting results of infants who were fed formula that was supplemented with 2′-FL (Table 1). These clinical experiences found that the supplementation of infant formula with 2′-FL is safe and well-tolerated [44,45], and that 2′-FL is absorbed and excreted with similar efficiency compared to 2′-FL in human milk [44]. In addition, infants who were fed formula with 2′-FL had immune benefits like the BF reference group [10], had fewer parent-reported infections, specifically respiratory infections, and had improved symptoms of formula intolerance in fussy infants. Therefore, adding 0.2 g 2′-FL/L to infant formula not only brings it closer compositionally to human milk, but also functionally. Additional clinical research may reveal other beneficial effects of 2′-FL in infant formulas, including other study populations such as preterm infants [51].

## Figures and Tables

**Table 1:** Summary of Clinical Experiences of Infants Fed 2′-FL Containing Formulas.

Study	Design	Population & Timeframe	Study Groups (Number Evaluable)	Results
Marriage et al., 2015 [44]	Prospective, randomized, controlled, growth, and tolerance study	420 healthy term infants Enrolled by 5 days of age and exited at 119 days of age.	CF with 2.4 g GOS/L (*n* = 68) Formula with 0.2 g 2‘-FL + 2.2 g GOS/L (*n* = 62) Formula with 1 g 2‘-FL + 1.4 g GOS/L (*n* = 59) BF reference group (*n* = 65)	There were NS differences for weight, length, and head circumference among the groups. All formulas were well tolerated (stool frequency, stool consistency, and incidence of spit up/vomit associated with feedings). There were NS differences in 2′-FL relative absorption and excretion among infants on the 2′-FL containing formulas and the BF infants.
Goehring et al., 2016 [10]	Cohort from Marriage et al. [44]	Cohort of 201 healthy term infants Enrolled by 5 days of age and blood drawn at 6 weeks of age.	CF with 2.4 g GOS/L (*n* = 39) Formula with 0.2 g 2‘-FL + 2.2 g GOS/L (*n* = 37) Formula with 1 g 2‘-FL + 1.4 g GOS/L (*n* = 37) BF reference group (*n* = 42)	Infants fed the 2′-FL containing formulas had 5 circulating cytokines concentrations that differed from infants that were fed the CF and did not differ from BF infants. PBMCs stimulated *ex vivo* with RSV had 2 cytokines from infants fed formula with 0.2 g 2′-FL/L that differed from the CF and were similar to BF infants, while formula with 1.0 g 2′-FL/L did not differ from CF, 0.2 g 2′-FL/L or from BF infants.
Kajzer et al., 2016 [45]	Prospective, randomized, multi-center, double-blinded, controlled tolerance study	131 healthy term infants Enrolled by 8 days of age and exited at 35 days of age.	CF without oligosaccharides (*n* = 30) Formula with 0.2 g 2‘-FL/L + 2 g scFOS/L (*n* = 35) BF reference group (*n* = 36)	2′-FL and scFOS containing formula was safe and well tolerated. There were NS differences among the three groups at 35 days of age, as evidenced by stool consistency, formula intake, anthropometric measures, and percent feedings with spit-up/vomit associated with feeding. BF infants had a greater number of stools/day than the formula fed infants.
Clinical Feeding Experience Study of a Partially Hydrolyzed Whey-Based Formula	Prospective, multi-center, single-arm study	59 healthy term infants identified as very or extremely fussy. Enrolled between 7–42 days of age and studied for 28 days.	Partially hydrolyzed whey-based formula with 0.2 g 2‘-FL/L + 1.8 g scFOS/L (*n* = 47)	2′-FL containing formula was safe and well tolerated by the fussy infants. Parents reported reduced severity of fussiness, amount of gassiness, number of hours of crying, and number of spit ups in fussy infants after 1 day of switching to 2′-FL containing formula, which was maintained throughout the 28 day study.
Post-Hoc Analysis of Adverse Events	Cohort from Marriage et al. [44]	Cohort of 205 healthy term infants Enrolled by 5 days of age and exited at 119 days of age.	CF with 2.4 g GOS/L (*n* = 101) Formula with 0.2 g 2‘-FL + 2.2 g GOS/L (*n* = 104)	Infants fed formula containing 0.2 g 2′-FL/L had fewer respiratory infections compared to CF.

Abbreviations: 2′-FL, 2′-fucosyllactose; AE, adverse event; BF, breastfed; CF, control formula; FF, formula-fed; GI, gastrointestinal; GOS, galactooligosaccharide; HMO, human milk oligosaccharide; NS, no significant; PBMCs, peripheral blood mononuclear cells; RSV, respiratory syncytial virus; scFOS, short-chain fructooligosaccharide.
